# Canadians’ Perceptions of Food, Diet, and Health – A National Survey

**DOI:** 10.1371/journal.pone.0086000

**Published:** 2014-01-23

**Authors:** Alyssa Schermel, Julio Mendoza, Spencer Henson, Steven Dukeshire, Laura Pasut, Teri E. Emrich, Wendy Lou, Ying Qi, Mary R. L’Abbé

**Affiliations:** 1 Department of Nutritional Sciences, University of Toronto, Toronto, Ontario, Canada; 2 Food, Agricultural, and Resource Economics Department, University of Guelph, Guelph, Ontario, Canada; 3 Department of Business and Social Sciences, Dalhousie University, Truro, Nova Scotia, Canada; 4 Division of Biostatistics, Dalla Lana School of Public Health, University of Toronto, Toronto, Ontario, Canada; Aga Khan University, Pakistan

## Abstract

**Background:**

Poor nutrition is harmful to one’s health as it can lead to overweight and obesity and a number of chronic diseases. Understanding consumer perceptions toward diet and nutrition is critical to advancing nutrition-related population health interventions to address such issues. The purpose of this paper was to examine Canadians’ perceived health and diet status, compared to their actual health status, and general concern about their own diet and beliefs about health. Also analyzed were some of the perceived barriers to eating “healthy” foods, with a focus on the availability of “healthy” processed foods.

**Methods:**

Two surveys were administered online to a group of Canadian panelists from all ten provinces during May 2010 to January 2011. Thirty thousand were invited; 6,665 completed the baseline survey and 5,494 completed the second survey. Panelists were selected to be nationally representative of the Canadian adult population by age, sex, province and education level, according to 2006 census data.

**Results:**

Approximately one third of Canadians perceived their health or diet to be very good while very few Canadians perceived their health or diet to be very poor. While the majority of Canadians believed food and nutrition to be very important for improving one’s health, fewer Canadians were concerned about their own diets. The majority of Canadians reported difficulty finding “healthy” processed foods (low in salt and sugar and with sufficient vitamins and minerals). Many also reported difficulty finding healthy foods that are affordable.

**Conclusion:**

Although consumers believe that nutrition is one of the most important factors for maintaining health, there are still a number of attitudinal and perceived environmental barriers to healthy eating.

## Introduction

Chronic diseases such as diabetes, heart disease, and cancer are the leading cause of death globally [1]. According to the World Health Organization, poor nutrition is one of four major modifiable behaviours (in addition to tobacco use, physical inactivity and the harmful use of alcohol) underlying most chronic diseases as it can lead to hypertension, hyperglycemia, hyperlipidemia, and overweight and obesity [1]. In addition to lowering quality of life and life expectancy, these risk factors dramatically increase health expenditures; in Canada, the annual economic burden of obesity alone has been estimated at $4.6 billion [2]. The health and economic impacts of poor nutrition on individuals and society provides a strong incentive to understand the factors that drive healthy and unhealthy food choices. Such information is essential for developing appropriate nutrition education campaigns, clear labelling strategies, and acceptable new products that will assist Canadians in improving their health and, at the same time, meet their needs for price, taste, convenience, and other drivers of food choice.

National consumer health surveys capture information on consumers’ knowledge, attitudes and behaviours to identify nutrition issues and aid in the development of healthy eating strategies. These issues must be tracked over time due to continual shifts in sociodemographics and changes in the food marketplace. In Canada, a number of major changes have occurred in recent years including the increased availability and use of pre-packaged convenience foods [3], the development of new and innovative “healthy” products [4], and the proliferation of “healthy” messages on the front of food packages [5], [6]. At the same time, the number of Canadians who are overweight or obese is rising [7]. Thus, in order to assess changes over time, information on nutrition knowledge, attitudes and behaviours, and health status is needed on a continual basis. Major Canadian surveys that provide information on health and nutrition include the Canadian Community Health Survey (CCHS), Cycle 2.2, Nutrition (2004) [8], National Population Health Survey (1994–95 and ongoing) [9], Provincial Nutrition Surveys (1989–1999) [10], and Tracking Nutrition Trends (TNT, 1989–2008) [11]. However, the available surveys are either out of date or provide only a narrow snapshot of nutrition knowledge, attitudes and behaviours, and other determinants of food choice in Canada. Furthermore, these sources do not allow the linking of this information with health status. For example, while the TNT surveys have been tracking nutrition knowledge, attitudes and behaviours for over 20 years, they do not provide data on the health status (BMI, chronic conditions) of participants. CCHS, on the other hand, provides detailed information on sociodemographics, health status and food intake, but does not capture nutrition attitudes and knowledge.

In the present paper we examine Canadians’ perceived health and diet status, compared to their actual health status, and general concern about their own diet and beliefs about health, using a nationally representative sample of Canadian adults. Also analyzed are some of the perceived barriers to eating “healthy” foods, with a focus on the availability of “healthy” processed foods.

## Methods

### Ethics Statement

The study is a collaboration among researchers from 11 universities across Canada led by the University of Guelph. Other participating universities include the University of Alberta, University of British Columbia, University of Manitoba, McGill University, Nova Scotia Agricultural College, University of Ottawa, University of Regina, Simon Fraser University, University of Toronto, and Wilfred Laurier University. Ethics approval was obtained prior to recruitment of panelists from the Research Ethics Boards of each of the above institutions. Additionally, all methods and tools were approved by the above Research Ethics Boards prior to administration.

Consent was obtained at two levels. At recruitment, potential panelists were asked orally if they agree to participate in the panel by the recruitment company. Written consent was not received due the large number of panelists invited to the study as well as the nature of the study being very low risk. Participants were informed that participation would be voluntary and that they could withdraw at any time. Personally identifiable information was obtained from the individuals agreeing to participate. This information included name, email address, and telephone number. Each panelist was assigned a unique identification number to permit linkage of data from all completed surveys. The name and email address of each respondent and the corresponding identification number were kept separately from the survey data and in encrypted form. With each survey, participants received a link to the consent form from the University of Guelph (File S1). This consent procedure was approved by the Research Ethics Boards of all universities involved in this study.

### The AFMNet Canadian Consumer Monitor Study

The Advanced Foods and Materials Network’s (AFMNet) Canadian Consumer Monitor (CCM) is a nationally representative consumer survey panel used to assess consumer attitudes towards food, diet and health, and to explore the acceptance of new product and process innovations directed at enhancing diet and health (e.g. probiotics, n-3 polyunsaturated fatty acids). The CCM research team, a diverse group of more than 20 researchers and graduate students, was divided into three working groups according to specific theme areas: consumer choice and information; food quality and ethical concerns; and food, diet and health.

### Sampling

In order to obtain a nationally representative sample of Canadians, panelists were recruited by age, sex, province (excluding Northern Canada), and education level, using the 2006 census data (Table 1) [12]. Surveys were conducted in English and French only (Canada’s two official languages). Eligible respondents were adults between the ages of 20 and 69 years inclusive, who were responsible (solely or partly) for their household’s food shopping and had access to a computer, Internet and email account. Individuals were excluded if their work was directly related to agriculture or the food industry.

**Table 1:** Demographic characteristics of CCM survey respondents (N = 6665), unweighted and weighted responses based on 2006 census data.

Characteristics	CCM, N (unweighted)	CCM, weighted* (%)	Census 2006(%)
**Sex**			
Male	2180	49.0	49
Female	4485	51.0	51
**Age group (years)**			
20–29	677	19.6	19.6
30–39	1155	20.4	20.4
40–49	1605	25.1	25.1
50–59	1908	21.4	21.3
60–69	1320	13.5	13.5
**Educational Level**			
Less than high school diploma	161	2.5	16.4
High school diploma	1458	21.3	25.8
Trades diploma	742	10.7	12.0
College diploma	2305	35.3	24.7
University diploma	1996	30.2	21.2
Missing	3	0	
**Province**			
Atlantic Canada			
New Brunswick	168	2.4	2.4
Newfoundland	158	1.7	1.7
Nova Scotia	393	2.9	2.9
Prince Edward Island	152	0.4	0.4
British Columbia	1051	13.2	13.2
Ontario	2334	38.3	38.3
Prairies			
Alberta	697	10.5	10.5
Manitoba	427	3.5	3.5
Saskatchewan	390	2.9	2.9
Quebec	895	24.4	24.4
**Household Income** †			
Less than $25,000	460	10.0	9.9
$25,000–49,999	994	18.0	20.9
$50,000–74,999	1070	19.5	22.3
$75,000–99,999	903	16.0	18.1
$100,000–124,999	652	11.5	12.0
$125,000–149,999	388	7.0	6.8
$150,000–174,999	280	5.0	3.8
$175,000–199,999	143	2.6	2.0
$200,000+	221	4.2	4.3
Missing	383	6.2	
**Occupation**			
White collar worker‡	3001	44.6	N/A
Blue collar worker§	1202	20.2	N/A
Student	325	7.9	N/A
Retired	1597	19.1	N/A
Unemployed	286	4.2	N/A
Missing	254	3.9	
**Marital Status**			
Married/Living with partner	4649	65.0	52.8
Single	999	21.2	32.3
Separated/Divorced	680	9.2	12.7
Widowed	176	1.8	2.3
Missing	161	2.8	
**Children below 18 living in the household**			
Yes	2240	35.9	N/A
No	4116	59.0	N/A
missing	309	5.1	
**Number of children**			
None/not indicated	4446	64.5	N/A
1	844	13.4	N/A
2	907	14.8	N/A
3+	468	7.3	N/A

CCM, Canadian Consumer Monitor.

* Responses were weighted according to the 2006 census data by age, sex, and province for those aged 20–69 and exclude Northern Canada.

† Data on household income was collected only in the 2^nd^ survey, where the number of respondents was 5,494.

‡ Includes occupations in management, business, finance, administrative, natural and applied sciences, health, social science, education, government service, religion, art, culture, recreation and sport.

§ Includes occupations in sales, service, trades, transport and equipment operators, primary industry, and processing, manufacturing and utilities.

### Recruitment

Panel recruitment was completed between September 2009 and January 2011 using the services of two companies specialized in online recruiting. Initial contact with potential panelists was made through a telephone screening interview. For those who met eligibility criteria and were willing to participate, contact information and information related to sampling requirements (age, sex, education level, and province) were collected and provided to the University of Guelph. The total number of contacts received was 31,223. Following recruitment, all communication with study panelists occurred via email from the University of Guelph.

### Survey Procedures

The CCM research team was responsible for designing and reviewing the survey questions prior to administration to panel members. Each survey underwent a plain language review, French translation, and pilot testing (when the survey instrument had not been tested before). Snap 10 Professional Survey Software (2010) (Snap Surveys Ltd., Portsmouth, NH) was used to electronically design the surveys for web administration. Companion software, Snap Webhost (2010), was used for survey publishing, response management (to monitor responses and send out timely reminders to panelists), and data collection.

An invitation to participate in each online survey was provided to each panelist through an electronic link sent by email. All 31,223 panelists were sent each survey link, regardless of whether or not they completed previous surveys. Panelists who did not complete the survey after the first email invite received up to three subsequent email reminders to complete the survey during the month. Panelists were asked to complete one 15- to 20-minute survey every 8 to 10 weeks, and had the option of completing the surveys in either English or French.

By August 2012, eleven surveys had been administered. The first two surveys reported in this paper covered a wide variety of food, health and nutrition topics (e.g. health and diet status, beliefs about health, perceptions about the food environment) and gathered demographic information to provide data for detailed analyses in later surveys. Questions were adapted from other national surveys including the Canadian Community Health Survey (CCHS) 2.2 (2004) [8], Tracking Nutrition Trends (TNT) VII (2008) [13] and the U.S. Food and Health survey (2010) [14] in order to include validated baseline questions which would enable us to compare our results with those from other national surveys.

### Statistical Analysis

Responses were weighted to be representative of the Canadian population by age, sex, and province. Survey weights were derived using a raking algorithm [15], [16], according to the 2006 census data. All statistical analyses were performed using the statistical software package Statistical Analysis System (SAS) version 9.2 (SAS Institute, Cary, NC).

## Results

### Respondents

Of the 31,223 contacts received, and after excluding those who provided little to no data in the baseline survey (n = 295), the total number of completions was 6,665 (completion rate of 21%). The number of completions for the second survey was 5,494 (completion rate of 18%). Although all 31,223 contacts were sent both surveys, some completed one survey but not the other. The number of respondents that completed both surveys was 4,104. Reasons for not completing the surveys included drop outs, faulty survey links, inability to open surveys, and rejection of emails as junk by security systems or firewalls.

Demographic characteristics of the respondents for the first survey in comparison with the 2006 census are presented in Table 1; characteristics for the second survey were similar to the first survey, and hence details are omitted. As commonly seen in other consumer surveys, some population groups were more likely to respond than others. For example, women were more likely to respond than men; persons of younger age and persons with lower education levels were less likely to respond. Results in this paper were therefore reported as weighted data, representative of the Canadian adult population in terms of sex, age, and province.

### Shopping and Cooking

Based on our sample, approximately half of Canadian adults (55%) reported doing more than 75% of the food purchasing for their household while about one quarter (28%) reported doing less than half (data not shown). Similarly, half reported doing more than 75% of the cooking for their household while nearly one third (31%) reported doing less than half.

### Health Status

Cancer and heart disease were reported in 5.5% and 3.9% of Canadian adults, respectively (Table 2). Diabetes was reported in 7.3% of Canadians, consistent with diabetes prevalence data from the National Diabetes Surveillance System (2004/05), which is based on diagnosis by a physician from health administrative records (7.1% of Canadians age 20+) [17]. Key risk factors including high blood pressure, high cholesterol, and obesity and overweight, associated with the development and progress of the above chronic diseases, were reported in approximately one fifth to one quarter of Canadians. High blood pressure was reported by 19.4% of Canadian adults, consistent with self-reported data in the 2007 CCHS (19.2% of Canadians age 20 years and older) [17].

**Table 2:** Self-reported medically diagnosed chronic conditions* of CCM respondents (N = 5,494), weighted to be representative of the Canadian population†.

Condition	N (unweighted)	All (%)†	Men (%)†	Women (%)†
Cancer	377	5.5	5.2	5.8
Diabetes	425	7.3	8.5	6.2
Heart Disease	238	3.9	4.8	3.0
Any GI disease	721	11.5	8.2	14.7
Arthritis and other immune disorders	1153	16.0	12.8	19.0
Osteoporosis	363	4.8	3.1	6.4
Hypothyroidism	451	6.2	2.9	9.3
Hyperthyroidism	150	2.4	1.4	3.3
Allergies and food intolerances	1340	22.5	17.9	26.9
High blood pressure	1233	19.4	21.6	17.4
Overweight/obesity	1580	25.4	22.6	28.1
High cholesterol	1120	18.4	22.9	14.2

CCM, Canadian Consumer Monitor.

* *Survey question:* Below we list a number of diseases or conditions. Have you ever been diagnosed with any of the following diseases or conditions? Please select YES or NO for each.

† Responses were weighted according to the 2006 census data by age, sex, and province for those aged 20–69 and exclude Northern Canada.

Some differences were observed between men and women. Generally, chronic conditions were self-reported by more women than men including any GI disease (reported by 6.5% more women), arthritis and other autoimmune disorders (6.2%), overweight and obesity (5.5%), hypothyroidism (6.4%), and allergies and food intolerances (9.0%). Compared to women, more men reported having high cholesterol (8.7%) and high blood pressure (4.2%).

### Perceived Diet and Health Status

**Figure 1:**
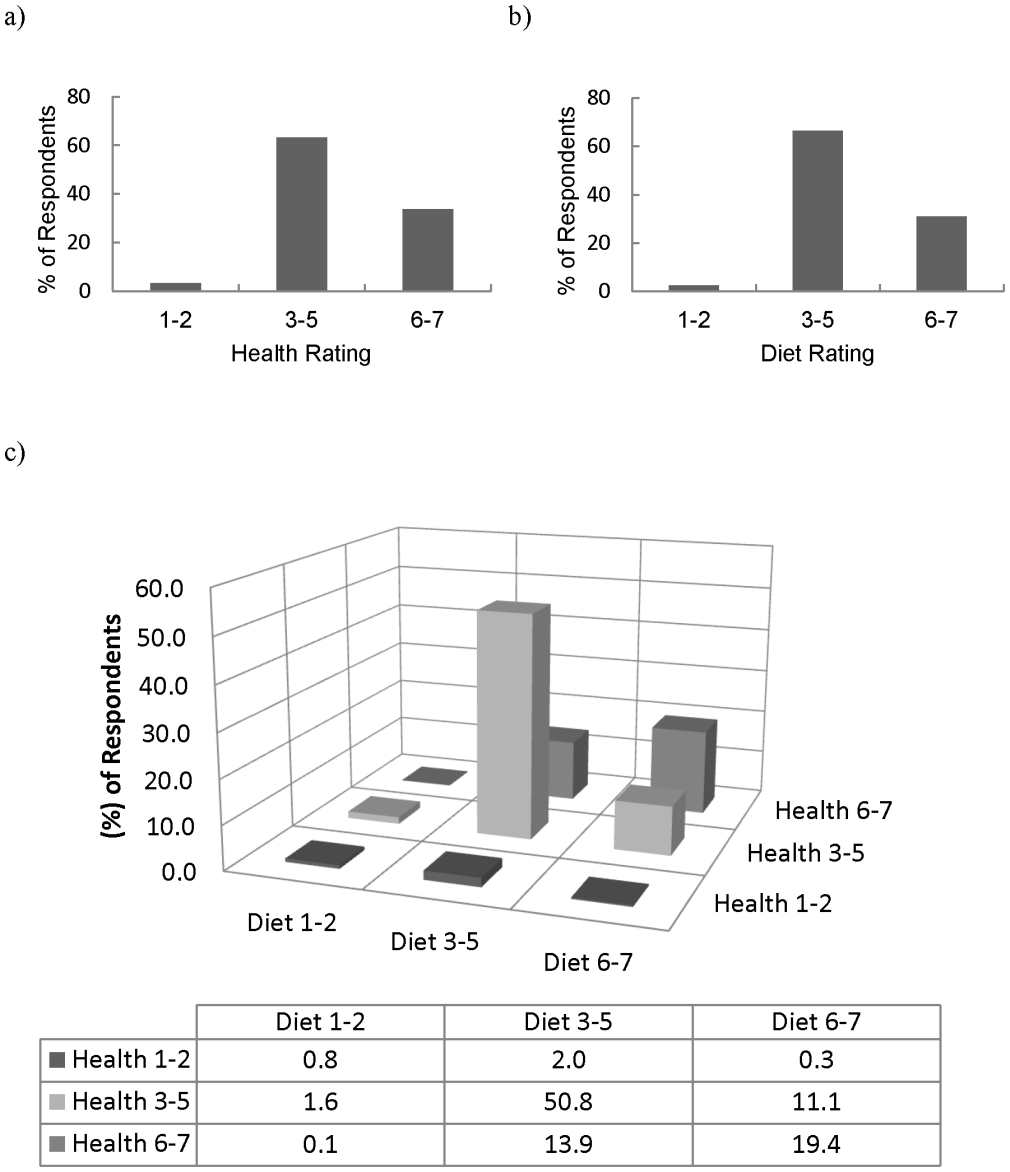
Self-reported ratings of overall diet and health status by CCM respondents* (N = 6,665). (a) Rating of overall health status†; (b) Rating of overall diet status‡; (c) Concordance of self-reported overall health status with diet status by respondent. *Respondents were weighted to be representative of the Canadian population by age, sex, and province according to the 2006 census data. †1 = very unhealthy; and 7 = very healthy; ‡1 = poor; and 7 = excellent; CCM: Canadian Consumer Monitor.

Approximately one third of Canadians (33.6%) perceived their health to be very good (6 or 7 rating on the 7-point Likert-scale) while very few (3.2%) perceived their health to be very poor (1 or 2 Likert-scale rating) (Figure 1(a)). Similar results were found regarding ratings of diet (Figure 1(b)).

The majority of Canadians (71.0%) rated both their diet and health similarly (those who rated their health as very unhealthy (1–2), neither very healthy nor very unhealthy (3–5), or very healthy (6–7) also rated their diet as poor, neither very good nor very bad, or excellent, respectively) (Figure 1(c)). Almost no Canadians (0.4%) rated their diet and health very differently (those who rated their health a 1–2 but their diet a 6–7, or vice versa).

### Main Factors Contributing to Health

**Figure 2:**
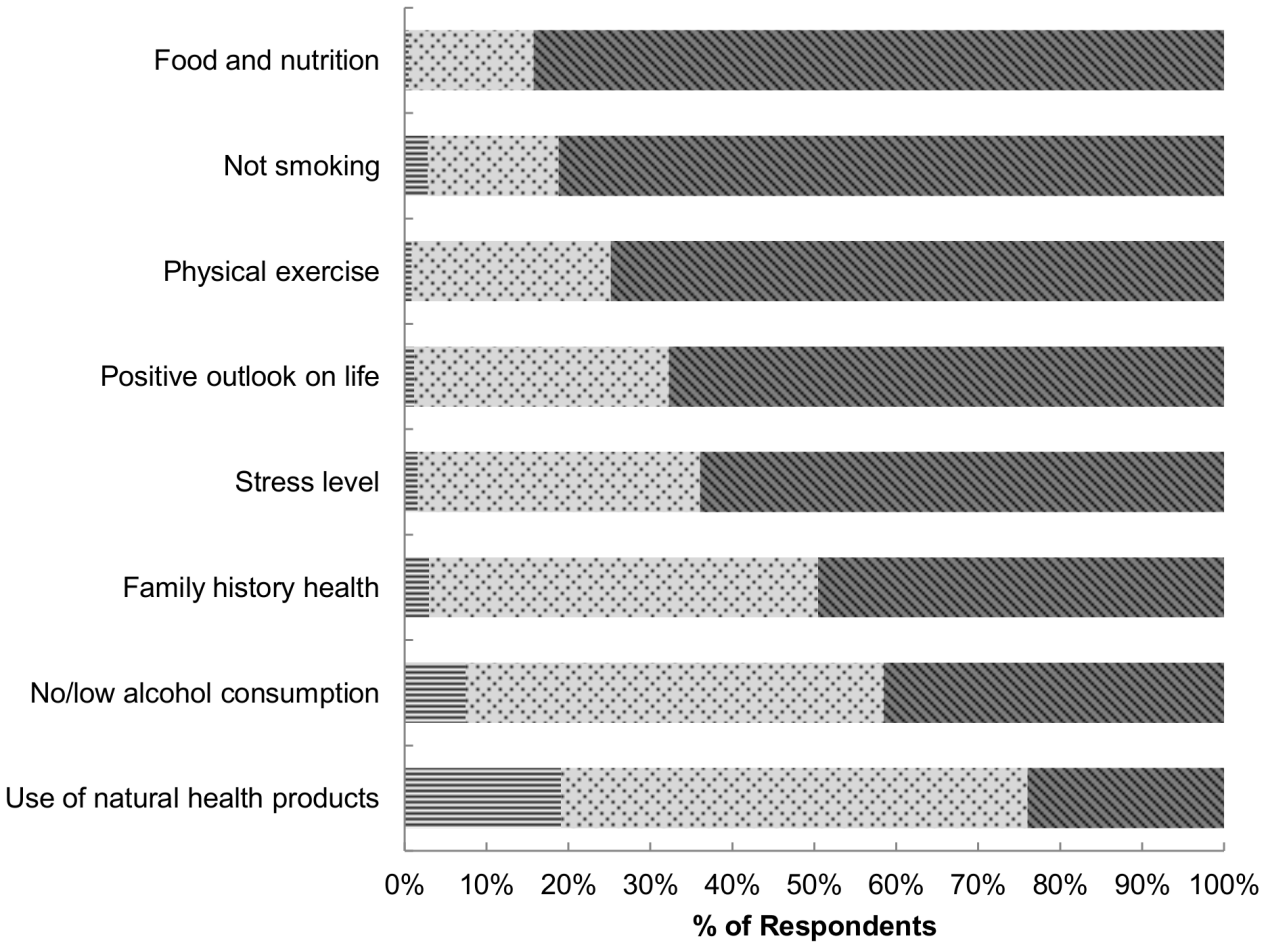
Perceived importance of factors related to maintaining or improving one’s own health* by CCM respondents† (N = 6,665). *1 = not at all important; and 7 = extremely important: <$>\raster="rg1"<$>1–2; <$>\raster="rg2"<$>3–5; and <$>\raster="rg3"<$>6–7. †Respondents were weighted to be representative of the Canadian population by age, sex, and province according to the 2006 census data. CCM: Canadian Consumer Monitor. Survey question: Using the following scale, please indicate how important you consider each of the following for maintaining or improving your own overall health. (Note: natural health products in Canada typically refer to herbal medicines or vitamin or mineral supplements).

The top three factors believed to be most important for maintaining overall health were food and nutrition (rated a 6 or 7 by 84.2% of Canadians), not smoking (81.2%) and physical exercise (74.9%) (Figure 2). Use of Natural Health Products (herbal medicines or vitamin or mineral supplements) was considered the least important factor (rated a 6 or 7 by only 24.0% of Canadians).

### Concern about Own Diet

The majority of Canadians (60.0%) were somewhat or extremely concerned about the healthfulness of their own diet (rated using the following scale: extremely unconcerned, somewhat unconcerned, neither concerned nor unconcerned, somewhat concerned, extremely concerned). Of these respondents, 64.3% had made an effort to change their eating habits (with the most common change being to reduce/stop or start eating certain foods) over the past two months. Of those who were “neither concerned nor unconcerned” or “unconcerned” (40.0% of respondents), 39.4% had still made an effort to change their eating habits.

### Attitudes Regarding the Availability of “Healthy” Foods Sold in Canada

**Figure 3:**
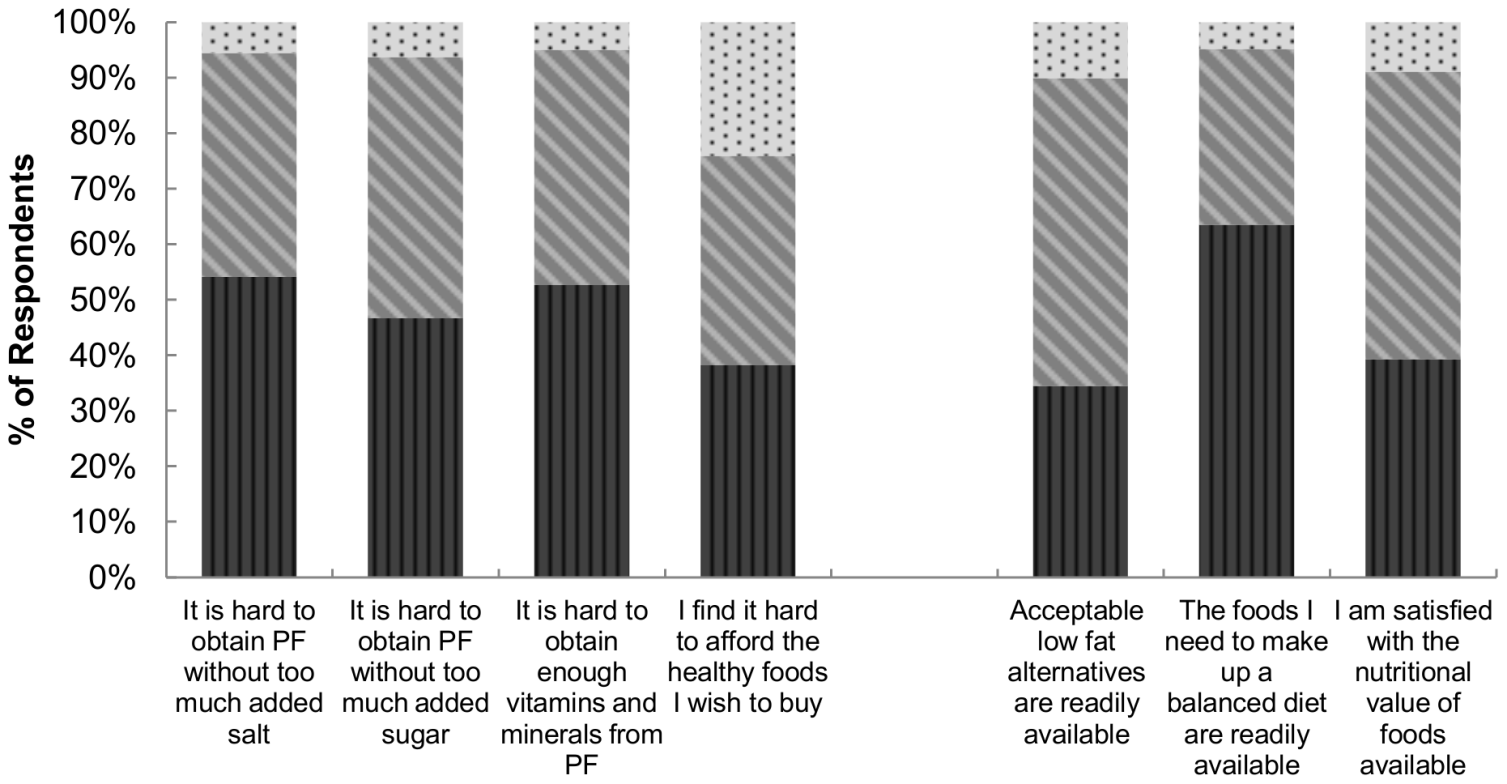
Perceptions of CCM respondents* (N = 6,665) regarding select statements about the availability of “healthy” foods sold in Canada†. *Respondents were weighted to be representative of the Canadian population by age, sex, and province according to the 2006 census data. †1 = completely disagree; and 7 = completely agree: 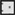
1–2; 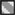
3–5; and 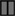
6–7. CCM: Canadian Consumer Monitor. PF: Processed Foods. *Survey question:* Please indicate the degree to which you agree or disagree with each of the following statements on the scales provided.

A large proportion of Canadians reported difficulty finding processed foods that are “healthy”; approximately half strongly agreed (6 or 7 rating) that it is hard to obtain processed foods without too much added salt (54.1%) or sugar (46.6%), or with enough vitamins and minerals (52.6%) (Figure 3). Similarly, only about one third (34.5%) strongly agreed that acceptable low fat alternatives are readily available.

Mixed responses were given regarding respondent satisfaction with the nutritional value of foods available. Despite these mixed ratings, the majority (63.5%) strongly agreed that the foods they need to make up a balanced diet are readily available to them. However, many respondents (38.2%) strongly agreed that it is hard for them to afford healthy foods.

## Discussion

The majority of Canadians (84%) believed food and nutrition to be very important for improving one’s health. However, fewer Canadians (60%) had translated this awareness into personal concern about diet. In addition, very few perceived their health or diet to be poor. Our findings are similar to those from the TNT VII survey, in which nearly one third of their sample population rated both their health and diet as “very good” or “excellent”, while very few respondents rated their health and diet as “poor”. Additionally, in this study, we examined barriers to consuming “healthy” foods which included price and difficulty finding “healthy” processed foods (i.e. low in salt and sugar and with sufficient vitamins and minerals).

It is worth noting that Canadians may be less than concerned with their diets because they perceive their diets and health to be better than they likely are. The present results point to a large discrepancy between Canadians’ perceptions of the healthfulness of their diets and their weight status compared to mean data from the CCHS 2.2. Indeed, only about one quarter of CCM respondents reported being overweight or obese. However, current rates of overweight and obesity based on measured weight and height from the CCHS indicate that approximately 59% of Canadians are overweight or obese [7]. This is more than double the self-reported frequency of overweight or obesity in our panel. However, this is not surprising as the CCHS data also show that Canadians significantly under-report their weight. Furthermore, data from the CCHS 2.2 showed that half of Canadian adults did not meet the minimum daily requirements for fruits and vegetables, over a quarter of Canadians aged 31 to 50 obtained more than 35% of their total calories from fat, and snacks accounted for more calories than breakfast and about the same number of calories as lunch [18]. This is in contrast with the large numbers in this survey who perceived their diets as healthy. Problematically, consumers who are satisfied with their diets may have lower motivation to change their diets compared to those who are less satisfied.

Our results also suggest that the food shopping environment may pose a barrier to eating healthy as many Canadians reported difficulty finding “healthy” processed foods and healthy foods that are affordable. These results support the need for interventions that lower the amount of “negative” nutrients such as sodium and sugar, and increase the amount of “positive” nutrients such as vitamins and minerals. Creating a healthy food environment that is lower in sodium is currently a national priority for improving the health of Canadians, as current intakes of sodium in Canada are more than double the recommended amount [19]. These results also support a need to educate consumers about the availability of healthy foods with a focus on price and identifying healthy processed foods. Subsidizing fresh healthy foods (e.g. fruits and vegetables) may be an option to help with affordability [2], considering that nearly 40% of respondents strongly agreed that the healthy foods they wish to buy are hard to afford.

This study is both innovative and novel as it is the first Canadian national survey to follow the same panel of consumers over time using multiple nutrition surveys. Most often in the field of market research, survey samples are drawn from pools of individuals that are pre-recruited for use in various, one time surveys. These pools are referred to as multi-purpose panels [20]. Unlike multi-purpose panels, our panel is unique to a particular collection of surveys, allowing for key information from the same respondents to be collected over time in multiple surveys. This not only makes it possible to cover a wide scope of subject matter but also to collect in-depth information on particular topics on a well-characterized study population. The inclusion of data on both nutrition knowledge, attitudes and behaviours as well as self-reported diagnosed conditions (Table 2) is a unique feature of this study. This information is being used in other studies by our research team to determine whether disease status affects nutrition knowledge, attitudes and behaviours (e.g. whether having hypertension affects certain food choice considerations related to sodium [21]).

The present study is limited by the underrepresentation of persons with low education levels and the exclusion of persons without access to computers, Internet and email accounts. According to the Canadian Internet Use Survey by Statistics Canada in 2009, 20% of the Canadian population aged 16 and over did not use the Internet [22]. As observed in other longitudinal surveys, the central challenge of the CCM may be respondent attrition as it is inevitable that over time, respondents will drop out. However, some panelists who did not respond to the first survey may respond to later surveys; and in fact some did. The CCM surveys hence allow for both cross-sectional and longitudinal analyses, although the latter will include fewer respondents since only those who participated in all relevant surveys can be linked for detailed analyses.

This study suggests that although consumers believe that nutrition is one of the most important factors for maintaining health, there are still attitudinal and perceived environmental barriers to healthy eating. If healthy eating strategies are to be effectively implemented, they need to address both the environmental and the person-related (e.g. attitudes and beliefs) determinants of healthy eating. The large volume of information collected using the CCM provides a wealth of information by which to analyze this complex interaction of factors influencing the knowledge, attitudes and behaviours of the modern Canadian consumer. Future research using the CCM can allow us to explore a number of diet and health relationships in a well characterized and large Canadian study population.

## Supporting Information

File S1 (DOCX)
