# Supplementary material for: Canadians’ Perceptions of Food, Diet, and Health – A National Survey
Source: PLoS One. 2014 Jan 23;9(1):e86000. doi: 10.1371/journal.pone.0086000 (PMC3900450; doi:10.1371/journal.pone.0086000)
Supplement: File S1 — (DOCX) [file pone.0086000.s001.docx]

**Canadian National Food Consumer Monitor**

Researchers in a number of Canadian universities, namely **University of Guelph, University of Toronto, McGill University, Nova Scotia Agricultural College, University of Manitoba, University of Regina, University of Alberta and Simon Fraser University**, are using a panel of consumers from across Canada for ongoing research on attitudes to food, diet and health. The panel is coordinated by Dr Spencer Henson at the University of Guelph. Funding for the panel is provided by the Foods and Materials Network (AFMNet), a Network of Centres of Excellence funded by the Government of Canada.

You have been asked to participate in the panel. As a participant in this panel you will be asked to complete a number of surveys throughout the year. These surveys will take around 20 to 30 minutes each to complete. You are free to participate or not in each survey and, should you choose not to participate, you can withdraw from a survey at any time. As well, you are free to skip any question you would prefer not to answer. By completing and submitting a survey, you provide consent to participate in the study.

Individual data from the study are only for academic research purposes and will not be passed on to anyone outside the research team. Verbatim quotes from answers to short written questions may be reported in an anonymous manner. Your responses to each survey are made in strict confidence, and your name will not be used in any report or data file. Your responses to each survey will be recorded using your randomly-assigned participant number. To protect your privacy, identifying information about yourself and participant identifying numbers will be stored in a locked cabinet in the project director’s office. All data collected in each survey will be stored on a server protected by password to which only members of the research team have access.

We do not judge that there are any notable risks from participation in the study. Further, your participation is important, as results from the study will inform policy-makers on issues related to food, diet and health, which ultimately may benefit consumers, such as you. You are not waiving any legal claims, rights or remedies because of your participation in this research study.

This study has been reviewed and received ethics clearance through the participating universities. If you have questions regarding your rights as a research participant, contact in the first instance:

Research Ethics Coordinator

University of Guelph

437 University Centre

Guelph, ON   N1G 2W1

Telephone: 519-824-4120 (x56606)

E-mail: sauld@uoguelph.ca

If you have any questions regarding the panel or broader study please do not hesitate to contact the project director:

Dr. Spencer Henson

Department of Food, Agricultural & Resource Economics

University of Guelph

Guelph ON N1G 2W1

Telephone: 519-824-4120 (x53134)

E-mail: [shenson@uoguelph.ca](mailto:shenson@uoguelph.ca)
